# Reversed paired-gRNA plasmid cloning strategy for efficient genome editing in *Escherichia coli*

**DOI:** 10.1186/s12934-020-01321-4

**Published:** 2020-03-10

**Authors:** Tingting Ding, Chaoyong Huang, Zeyu Liang, Xiaoyan Ma, Ning Wang, Yi-Xin Huo

**Affiliations:** 1 Key Laboratory of Molecular Medicine and Biotherapy, School of Life Sciences, Beijing Institute of Technology, No. 5 South Zhongguancun Street, Haidian District, Beijing, 100081 China; 2 SIP-UCLA Institute for Technology Advancement, Suzhou, 215123 China

**Keywords:** Plasmid stability, Paired gRNAs, Direct repeats, Inverted repeats, Large genomic deletion

## Abstract

**Background:**

Co-expression of two distinct guide RNAs (gRNAs) has been used to facilitate the application of CRISPR/Cas9 system in fields such as large genomic deletion. The paired gRNAs are often placed adjacently in the same direction and expressed individually by two identical promoters, constituting direct repeats (DRs) which are susceptible to self-homologous recombination. As a result, the paired-gRNA plasmids cannot remain stable, which greatly prevents extensible applications of CRISPR/Cas9 system.

**Results:**

To address this limitation, different DRs-involved paired-gRNA plasmids were designed and the events of recombination were characterized. Deletion between DRs occurred with high frequencies during plasmid construction and subsequent plasmid propagation. This recombination event was RecA-independent, which agreed with the replication slippage model. To increase plasmid stability, a reversed paired-gRNA plasmids (RPGPs) cloning strategy was developed by converting DRs to the more stable invert repeats (IRs), which completely eliminated DRs-induced recombination. Using RPGPs, rapid deletion of chromosome fragments up to 100 kb with an efficiency of 83.33% was achieved in *Escherichia coli*.

**Conclusions:**

The RPGPs cloning strategy serves as a general solution to avoid plasmid RecA-independent recombination. It can be adapted to applications that rely on paired gRNAs or repeated genetic parts.

## Background

CRISPR-based systems are powerful tools for genetic manipulations in both eukaryotic and prokaryotic organisms, which solely rely on single guide RNA molecule (gRNA) for targeting [1, 2]. The versatility of CRISPR systems can be greatly enhanced when two distinct genomic loci are targeted simultaneously. For example, paired gRNAs are required to dramatically reduce off-target mutations [3], to achieve combinatorial genome modifications [4] and to facilitate large genomic deletion [5, 6]. The stable co-expression of paired gRNA determines the precision, the reliability, and the efficiency of CRISPR applications.

Although various cloning strategies have been established for the expression of paired or multiple gRNAs, the instability of gRNA plasmids is still an urgent problem to be solved. In general, multiple targeting spacers can be expressed via the CRISPR array or gRNA cassettes. However, plasmids with direct repeats (DRs) are difficult to assemble in vitro and are also genetically unstable in vivo. When using CRISPR array, recombination between DRs of the array caused the loss of spacer sequences and the subsequent failure of genomic modification [2, 5]. Similarly, co-expressing multiple gRNAs in one plasmid resulted in self-homologous recombination if gRNA cassettes were transcribed by independent but identical promoters in the same direction [7–9]. To eliminate recombination, the construction of promoter library and functional gRNA scaffold library could be further explored [10], but it may be limited by optional constructive promoters and complex machine learning technology. Thus, we attempted to investigate the recombination mechanism of DRs-involved paired-gRNA plasmids and develop simple but universal cloning strategies to prevent DRs-mediated recombination genetically.

Normally, the rearrangements of DRs are induced by recombinational or replicational mechanism in *E. coli* [11]. The former is mainly mediated by recombinase A (RecA) which promotes the pairing and the strand exchange between homologous sequences to form heteroduplex DNA [12–14]. The RecA protein is essential in most types of homologous recombination events, but not in RecET pathway-dependent circular and linear DNA recombination [15]. RecA-dependent recombination produces intermolecular interactions and appears to require a threshold of minimal homology [16]. For homologies less than 200 bp in length, the plasmid rearrangement can occur in a RecA-independent manner [17–21]. This type of rearrangement is enhanced by replication errors that hinder the DNA replication process [22]. The proximity of the repeat sequences also plays an essential role, presumably because the two repeats must interact within a single replication fork [23].

Depending on plasmid recombination products and the inverting sequences between DRs, three mechanisms of RecA-independent rearrangements were presented: slipped misalignment, sister-chromosome slipped misalignment, and single-strand annealing [16]. The replication slippage model explains the homology and proximity dependence of RecA-independent rearrangements that result in plasmid deletion or expansion. In addition, the sister-chromosome slipped misalignment model is proposed for RecA-independent plasmid dimers. The single-strand annealing model is well documented in eukaryotes for RecA-independent deletion formation associated with palindromes, but it is inefficient in wild type *E. coli* strains. However, the recombination mechanism of plasmids expressing paired gRNAs remains unknown, and further researches are needed.

Here, we investigated the deletion mechanism of DRs-involved paired-gRNA plasmids, including the effects of the transformation methods, culture media and plasmid architectures on paired-gRNA plasmids stability. The recombination events during plasmid construction and subsequent propagation could not be eliminated in the presence of DRs. To eliminate DRs, a simple reversed paired-gRNA plasmids (RPGPs) cloning strategy was developed by placing paired gRNA cassettes in the opposite direction, which converted DRs to invert repeats (IRs). RPGPs could thoroughly avoid DRs-mediated recombination during DNA replication and achieve large genomic deletion with high efficiency. Thus, RPGPs have a great potential to facilitate the construction of large-scale gRNA libraries for CRISPR applications that require paired gRNAs. The reversed-assembly cloning strategy can also be applied to the construction of other plasmids containing repeated genetic parts.

## Results

### The stability of DRs-involved paired-gRNA plasmids pDG-A-X series

**Fig. 1:**
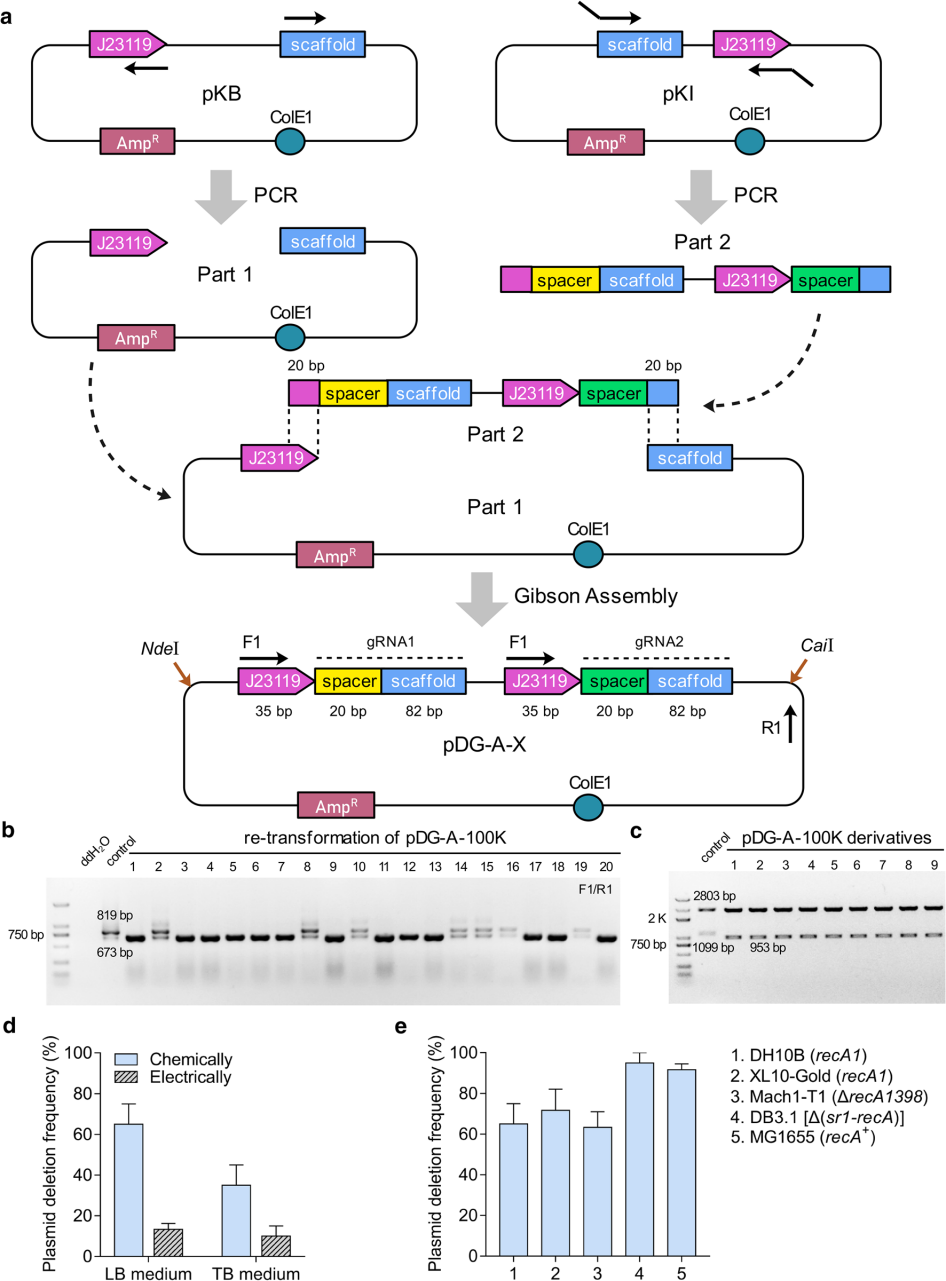
The design and stability of DRs-involved paired-gRNA plasmids pDG-A-X in *E. coli*. **a** The modular construction strategy of pDG-A-X series. pKB plasmid was used for PCR amplification of DNA part 1, which contained pDG-A-X backbone, one constitutive promoter J23119, and a gRNA scaffold. pKI plasmid was used for PCR amplification of part 2 series, which contained a gRNA fragment followed by another promoter J23119 and 20-bp space sequence. For the PCR reaction, the 20-bp space sequences specific for two targeted loci and the 20-bp overlap sequences for assembly were embedded in primers as a part of insert. Gibson Assembly method was used to assemble these parts into pDG-A-X series. **b** Representative PCR results for the deletion frequencies of pDG-A-100K after re-transformation. **c** The double restriction enzyme digestion analyses of pDG-A-100K and its deletion derivatives. **d** Effects of transformation methods and culture media on plasmid deletion frequency. **e** The plasmid deletion frequencies of pDG-A-100K after the re-transformation into various strains of *E. coli*. Values and error bars represent the mean and the s.d. (n = 3)

To study the stability of DRs-involved paired-gRNA plasmids, pDG-A-X series for co-expression of two gRNAs were employed. A functional gRNA contains a 20-bp spacer sequence for targeting and a 82-bp scaffold that binds Cas9 protein [4]. Each gRNA was transcribed by a 35 bp constitutive promoters J23119 (Fig. 1a). Plasmid rearrangements were detected by PCR primers F1/R1 after plasmid construction and re-transformation.

Plasmid pDG-A-100K for 100-kb genomic deletion was constructed by using *E. coli* DH10B strain as host. However, the deletion frequency of 73.33% was observed after pDG-A-100K plasmid construction. Similarly, the plasmid deletion frequency was around 65% after re-transformation of the correct pDG-A-100K (Figs. 1b, 4b). PCR results indicated that the deletion occurred between the paired-gRNA regions of these mutant plasmids. Subsequent DNA sequencing results demonstrated that one of two gRNAs along with its promoter was eliminated. Furthermore, the double restriction enzyme digestion analyses by using *Nde*I and *Cai*I showed the deletion only occurred between the paired-gRNA regions, rather than other parts of plasmids (Fig. 1c).

To enhance the stability of pDG-A-100K, the effects of experimental conditions including DNA transformation methods and culture media were then assessed during plasmid re-transformation in DH10B strain. Compared with transformation by heat shock, electrotransformation led to a 5.6-fold decrease in the plasmid deletion frequency for cells cultured in LB medium and a 3.5-fold decrease for cells cultured in TB medium (Fig. 1d). The nutrient supplies for plasmid propagation also influenced its stability. Replacing the LB medium with the nutrient-rich TB medium reduced the plasmid deletion frequency by half when DNA was chemically transformed into cells, while no significant decrease in the plasmid deletion frequency was achieved when plasmids were transformed electrically (Fig. 1d). Therefore, pDG-A-100K appeared to be more stable when introduced into cells by electroporation and propagated in rich medium, but neither could eliminate the events of plasmid rearrangement.

### The patterns of plasmid rearrangements

**Fig. 2:**
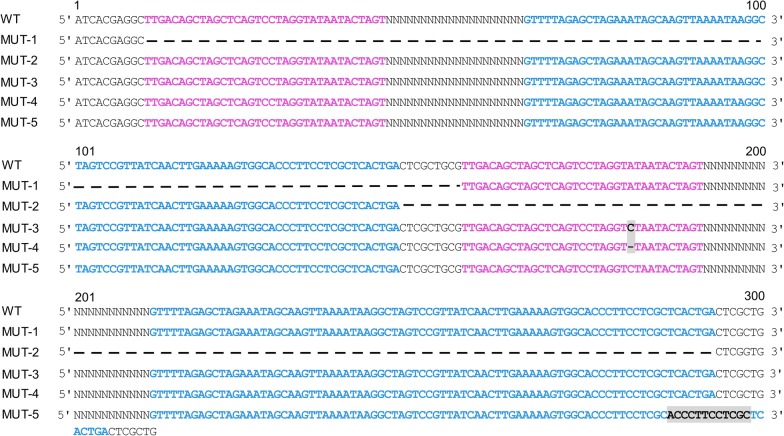
Representative DNA sequencing results of recombination derivatives from pDG-A-X series. MUT-1 had deletion of the first gRNA expression cassette; MUT-2 had deletion of the second gRNA expression cassette; MUT-3 and MUT-4 had point mutations in the second promoter J23119; MUT-5 had 12-bp repeated insertion in the second gRNA scaffold. Promoter J23119 (purple), 82-bp scaffold (blue) and the mutated sequence (highlighted) are denoted. The strikeout represents deleted sequence

Various recombination derivatives were discovered during DRs-mediated recombination events of pDG-A-X series (Fig. 2). Based on our observations, two main types of deletion were summarized: the deletion of the first gRNA expression cassette along with its promoter (MUT-1), and the deletion of the second gRNA expression cassette together with its promoter (MUT-2). Among these observations, MUT-1 and MUT-2 never appeared simultaneously in tests of pDG-A-X series. Point mutations and insertions also occurred during plasmid construction and subsequent propagation. For example, point mutations in the –10 regions (MUT-3/4) or –35 regions of promoter J23119 appeared frequently, which could affect the transcription process of gRNAs. A 12-bp repeated insertion at the end of the gRNA scaffold was also detected (MUT-5), which could influence the normal structure of gRNA. Taken together, random deletion of one of the paired gRNA expression cassettes dominated the recombination events for pDG-A-X series, with other spontaneous mutations occurred between the paired-gRNA regions, all contributing to instability of the paired-gRNA plasmids.

### The RecA dependency of paired-gRNA plasmids recombination

To test whether the recombination of pDG-A-X series relied on the RecA enzyme, the correct pDG-A-100K was re-transformed into various *E. coli* strains with the genotypes of *recA1*, Δ*recA1398*, Δ(*sr1*-*recA*) or *recA*^+^ (Fig. 1e). Compared with wild-type *recA*, *recA1* has a G to A point mutation at position 482 and can no longer mediate recombination [24]; Δ*recA1398* encodes a truncated non-functional RecA mutant; and Δ(*sr1*-*recA*) represents complete deletion of *recA*. Thus, there is no functional RecA protein in DH10B *recA1*, XL10-Gold *recA1*, Mach1-T1 Δ*recA1398* and DB3.1 Δ(*sr1*-*recA*) strain. In these *recA* mutant strains, DRs-induced recombination occurred with the frequencies of 63.33–95%. No distinct difference in plasmid deletion frequency was found for DH10B, XL10-Gold and Mach1T1 strain, while the plasmid deletion frequency even increased up to 95% for DB3.1 strain. As a wild-type laboratory strain, MG1655 *recA*^+^ strain expresses functional RecA protein, and the plasmid deletion frequency in MG1655 was also up to 91.67%. All results indicated that RecA-independent recombination played a great role on the deletion of pDG-A-X series in *E. coli*.

### The optimized replication slippage model for plasmid rearrangements

Owing to the dominance of plasmid deletions rather than plasmid dimers in the recombinant products, the replication slippage model for RecA-independent recombination best suited this recombination events. This model suggested that during replication, the slipped nascent strand could form a loop within the template or the nascent strand to facilitate deletion or expansion [16, 25]. As for the deletion process, it was predicted that the DNA polymerase may arrest and dissociate from the DNA sequence, allowing the nascent strand containing the first copy of the DRs to separate from its template strand. Meanwhile, a loop structure formed between the DRs on the template strand brings the two repeated regions closer, facilitating the nascent strand to translocate and pair to the second copy of the DRs. As a result, the deletion of one entire copy of DRs occurs when DNA synthesis resumes [16]. Moreover, replication slippage is thought to occur on single-stranded DNA template and therefore happens more frequently during lagging-strand synthesis, as the lagging strand template is single-stranded [22].

**Fig. 3:**
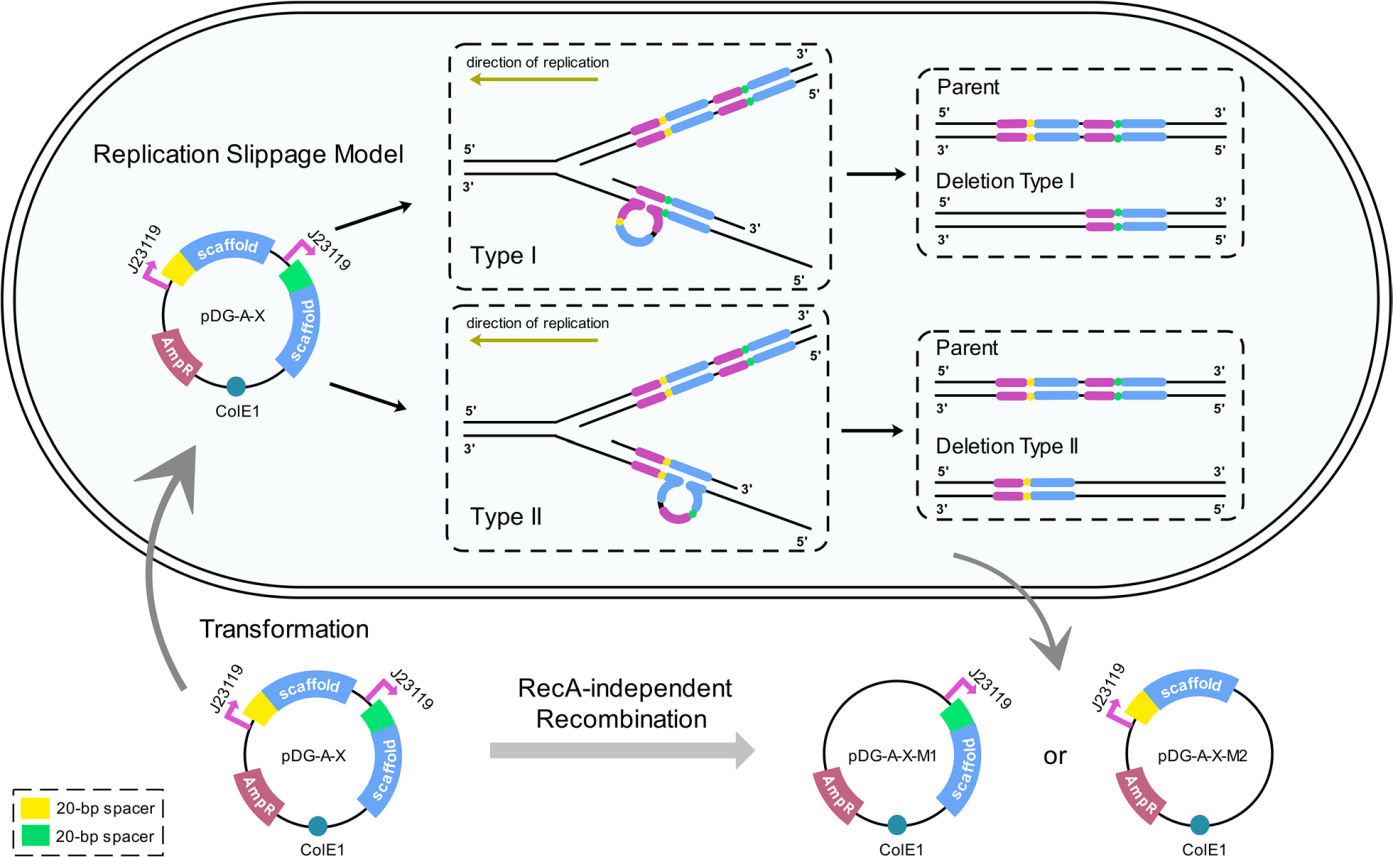
The deletion mechanism of pDG-A-X series during DNA replication. Plasmid pDG-A-X was designed for double gRNA expression: gRNA1 and gRNA2. Each gRNA containing a 20-nt spacer sequence (yellow or green) and an 82-bp scaffold (blue) was transcribed by a constitutive promoter J23119 (purple). During the DNA replication process in *E. coli*, pDG-A-X series generated the Types I or Type II slipped misalignment of the Okazaki fragment, which formed a loop within the lagging strand template to facilitate the formation of deletion. Deletion Type I: When the second promoter J23119 of the template strand was employed as mispaired position, the deletion of the first gRNA expression cassette occurred, leading to the formation of pDG-A-X-M1. Deletion Type II: When the repeated gRNA scaffold mediated the plasmid recombination, the second gRNA expression cassette of pDG-A-X series was deleted to form pDG-A-X-M2. The golden arrows indicate the direction of plasmid replication

According to the two types of deletion of pDG-A-X series, the optimized replication slippage model of the pDG-A-X series was proposed (Fig. 3). The ColE1 origin of replication (ORI) [26] of pDG-A-X series, a high-copy-number replicon, determines unidirectional replication as indicated by golden arrows in Fig. 3. During replication, pDG-A-X series generated the Types I or Type II slipped misalignment of the Okazaki fragment to facilitate the formation of deletion. When the second promoter J23119 of the template strand was employed as mispaired position, the deletion of the first gRNA expression cassette occurred, leading to the formation of pDG-A-X-M1. When the repeated gRNA scaffold mediated the plasmid recombination, the second gRNA region was deleted to form pDG-A-X-M2.

### Effects of promoters and ORIs on pDG-A-X series stability

**Fig. 4:**
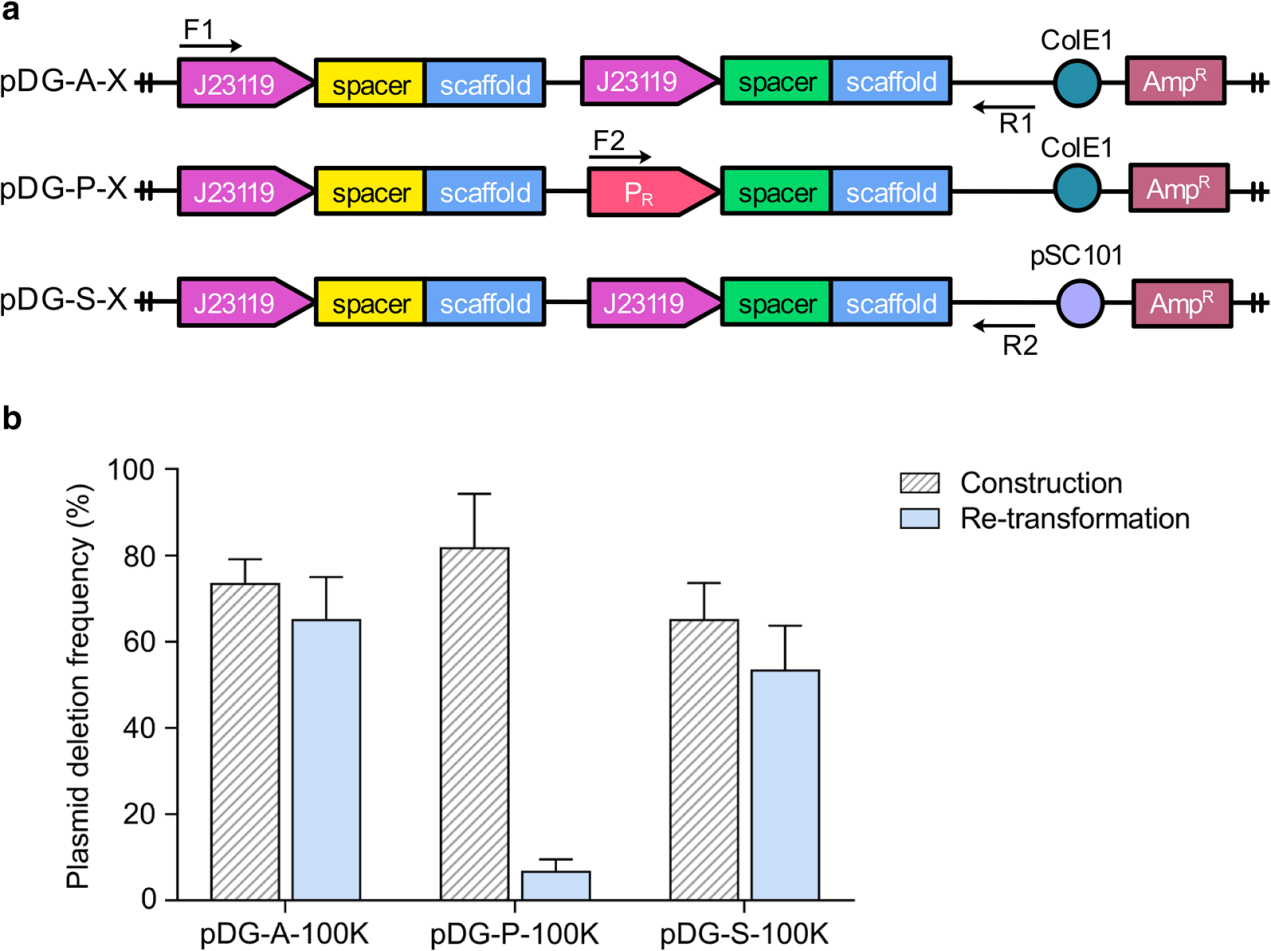
The comparisons of stability of DRs-involved paired-gRNA plasmids pDG-A-X, pDG-P-X and pDG-S-X series. **a** Diagrams of pDG-A-X, pDG-P-X and pDG-S-X series. **b** The plasmid deletion frequencies of pDG-A-X, pDG-P-100K and pDG-S-100K during plasmid construction and re-transformation processes. Values and error bars represent the mean and the s.d. (n = 3)

The effect of different plasmid architectures on plasmid stability was then evaluated. As shown in Fig. 1, there were two pairs of DRs in pDG-A-X. One was the repeated 35-bp J23119 promoters while the other one was the repeated 82-bp gRNA scaffolds. To reduce the number of DRs, pDG-P-X was designed by replacing the second promoter J23119 with an alternative 49-bp P_R_ promoter (Fig. 4a). After the assembly products of pDG-P-100K were introduced into DH10B strain, the deletion frequency of pDG-P-X was up to 81.67% when verified by primers F1/R1 (Fig. 4b). These deletion derivatives of pDG-P-100K didn’t contain promoter P_R_ region when verified by primers F2/R1. The following DNA sequencing demonstrated that pDG-P-100K generated spontaneous deletion of the second gRNA region to form pDG-A-100K-M2. Although it was difficult to obtain correct pDG-P-X series plasmids by Gibson Assembly method, these plasmids could be more stably maintained after re-transformation once the correct plasmid was obtained firstly **(**Fig. 4b).

To further investigate the effect of copy number on plasmid stability, pDG-S-X series were designed by replacing the high-copy-number ColE1 ORI with pSC101, a low-copy-number ORI (< 8 copies/cell) [27] (Fig. 4a). However, pDG-S-100K still had high deletion frequencies of 65% and 53.33% during plasmid construction and re-transformation (Fig. 4b). These results demonstrated that changing the promoter or the ORI of DRs-involved paired-gRNA plasmids pDG-A-X series didn’t eliminate the events of plasmid rearrangement.

### Design of RPGPs cloning strategy

**Fig. 5:**
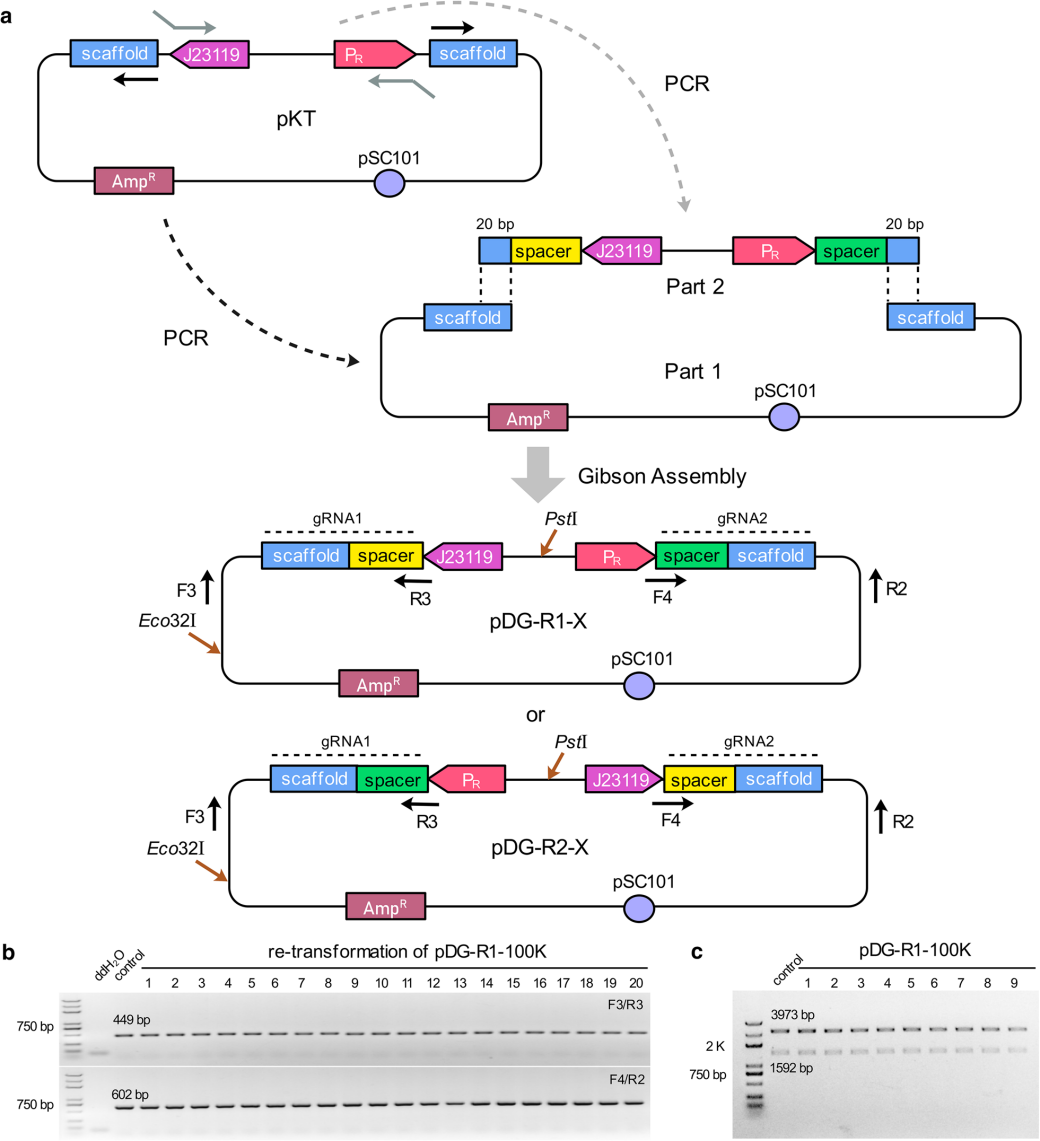
The design and stability of RPGPs pDG-R-X in *E. coli*. **a** The modular construction strategy of pDG-R-X series. pKT plasmid was designed for PCR amplification of DNA part 1 and part 2 series. DNA part 1 which contained pDG-R-X backbone and two reversed repeated gRNA scaffolds was amplified by using only one prime. DNA part 2 contained two different promoters followed by a 20-bp spacer sequence, respectively. For the PCR reaction, the 20-bp spacers specific for two targeted loci and another 20-bp overlap sequences for assembly were embedded in primers as a part of insert. Gibson Assembly method was used to assemble these parts into pDG-R1-X or pDG-R2-X series. **b** Representative PCR results of pDG-R1-100K after re-transformation. **c** The double restriction enzyme digestion analyses of pDG-R1-100K

In attempt to avoid DRs-induced plasmid rearrangements genetically, a reversed paired-gRNA plasmids (RPGPs) cloning strategy was developed to construct pDG-R-X series (Fig. 5a). Compared with pDG-A-X, the plasmid architectures of pDG-R-X were modified through changing the promoter of the second gRNA, the ORI, and the direction of gRNA cassettes. Two gRNA cassettes were placed in opposite directions with one expressed by J23119 promoter and another by P_R_ promoter, thus turning the two 82-bp gRNA scaffolds into inverted repeats (IRs). Moreover, two different promoters ensured the order of the two 20-bp spacers, when the spacer and the 20-bp overlap sequences for assembly were embedded in primers as a part of insert. Since the overlap sequences were repeated but reversed, the insert could be assembled in two directions, leading to the formation of pDG-R1-X or pDG-R2-X (Fig. 5a). As we expected, pDG-R-100K didn’t generate any plasmid rearrangement events during plasmid construction process, when verified by PCR reactions (F3/R3 and F4/R2) and DNA sequencing.

To further examine the stability of PRGPs, the correct pDG-R1-100K plasmid was retransformed into DH10B strain and verified by PCR reaction. All of 50 colonies produced a 449-bp and a 602-bp band when amplified by primer pairs F3/R3 and F4/R2, respectively. Representative colony PCR results are shown in Fig. 5b. Nine of corresponding plasmids were digested by *Eco*32I and *Pst*I and produced two bands with correct sizes of 3973 bp and 1592 bp (Fig. 5c). The following DNA sequencing results also confirmed that pDG-R1-100K maintained the intact paired gRNA expression cassettes without any mutations.

### Large genomic deletion mediated by RPGPs

**Fig. 6:**
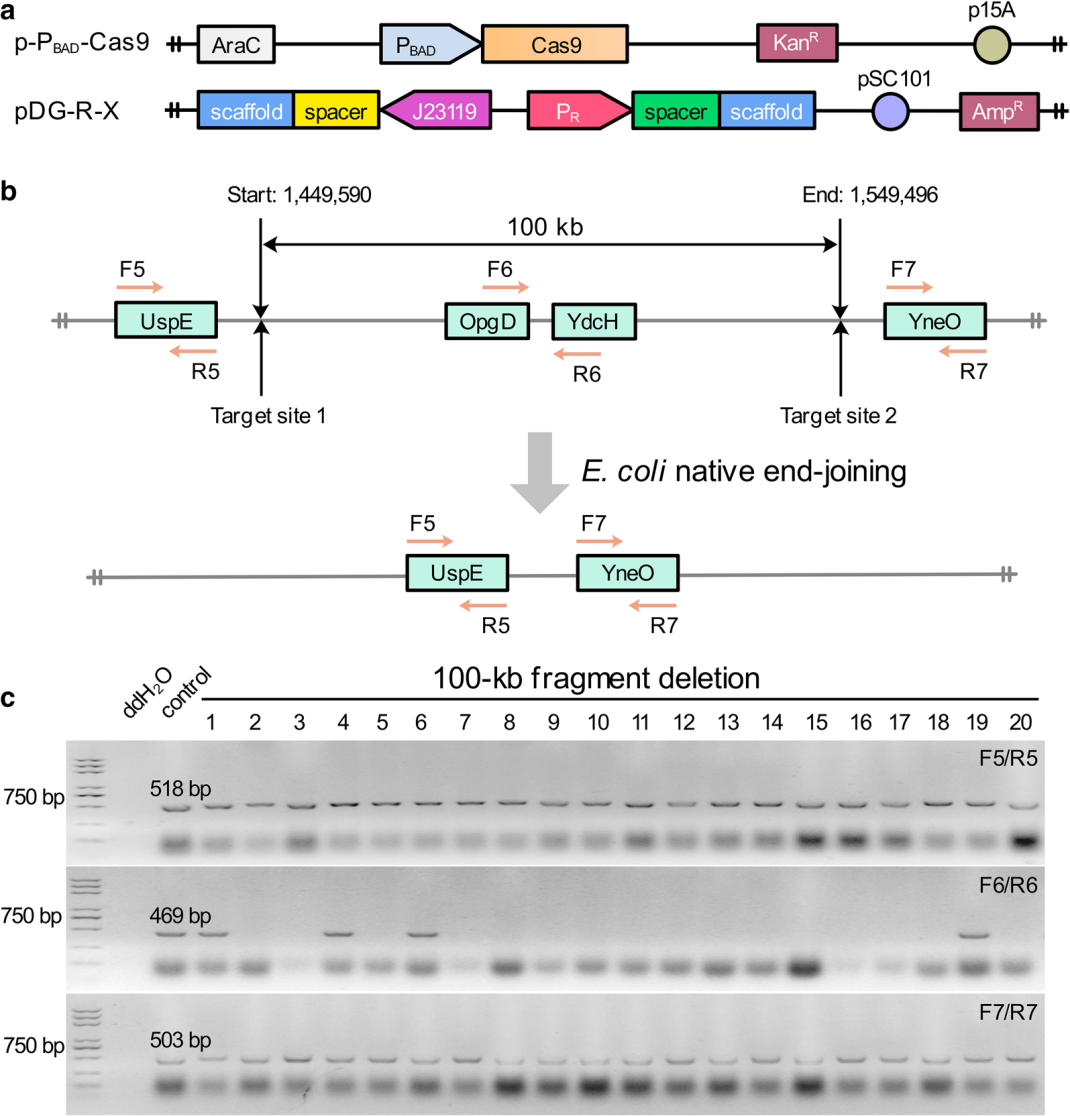
The RPGPs-assisted CRISPR/Cas9 system for the 100-kb fragment deletion in *E. coli*. **a** Diagrams of p-P_BAD_-Cas9 and RPGPs pDG-R-X series for genomic deletion. p-P_BAD_-Cas9 plasmid was used to express Cas9 protein induced by L-arabinose. pDG-R-X was used to express paired gRNAs without inducer. **b** Deletion of 100-kb genomic fragment in *E. coli*. **c** Representative PCR results of a 100-kb fragment deletion. Colonies were randomly picked for PCR screening, and a wild-type colony served as control

To test the practicability of RPGPs, RPGPs-associated CRISPR/Cas9 system was used for large genomic deletion in *E. coli* MG1655 strain. Since the double-strand breaks (DSBs) in *E. coli* can be repaired through its native end-joining mechanism [28], two plasmids were required for editing: p-P_BAD_-Cas9 plasmid contained p15A ORI, a *kan* gene, and a *cas9* gene under control of the arabinose-inducible araBAD promoter (P_BAD_); RPGPs pDG-R-X series contained pSC101 ORI, a *bla* gene and paired-gRNA expression cassettes (Fig. 6a). Cas9 used here was an evolved SpCas9 variant xCas9-3.7 [29], which could reduce the survival rate of wild-type cells and increase the positive rate of large genomic editing.

Plasmid pDG-R1-100K was applied to coexpress two gRNAs for the deletion of a 100-kb nonessential fragment from the *E. coli* chromosome (1,449,590-1,549,496) (Fig. 6b). The targeting sequences of 100-kb fragment were summarized in Additional file 1: Table S2. Since the native end-joining mediated DNA repair resulted in genomic deletion of random length around two targeted loci, we designed three pairs of primer F5/R5, F6/R6, and F7/R7 to check positive mutants among forty randomly selected colonies, and representative PCR results are shown in Fig. 6c. Approximately, 83.33% editing efficiency was achieved in this test, while negative colonies (16.67%) in the experimental group were also obtained. Further investigation showed that these negative colonies were not the wild type, but contained sequence deletion of stochastic length in the two target sites. The results indicated that RPGPs-associated CRISPR/Cas9 system was successfully used for large genome editing in *E. coli*.

## Discussion

The versatility of CRISPR systems can be greatly enhanced when two distinct genomic loci are targeted simultaneously, which could enable combinatorial genome modifications and large genomic deletion, and reduces off-target mutations [3–6]. As for these applications, effective gene editing requires the stable co-expression of paired gRNA. However, the DRs-involved paired-gRNA plasmids are susceptible to self-homologous recombination, causing the loss of spacer sequences and the subsequent failure of genomic modification. In this study, we demonstrated that elimination of DRs was the key to avoid RecA-independent recombination of the paired-gRNA plasmids. By simply placing paired gRNA cassettes oppositely, or in other words, by transforming DRs into IRs, deletion events of paired-gRNA plasmids were completely eliminated. This strategy made it easy to obtain and maintain the correct plasmids expressing paired gRNAs, and thus could be employed in applications that rely on paired gRNAs or repeated genetic parts.

The RecA protein was shown to be essential in most types of homologous recombination event, while RecBCD, RecJ or RecQ protein could process the dsDNA to produce ssDNA suitable for RecA function [15, 16, 30]. Although the RecA-involved RecBCD pathway is the major pathway for recombination in wild type cells, several of plasmid or genomic recombination events introduced by short DRs also occurred in manners independent of RecA protein [17–21]. Both RecA-dependent and RecA-independent recombination are homology-driven, with the former relies on the length of DRs more than 200 bp [17]. In this study, the recombination of pDG-A-X series was RecA-independent, probably because the length of two pairs of DRs are just 35 bp and 82 bp, respectively.

Since the RecA-independent recombination involved with different replication mechanisms could produce multiple products, the recombination derivatives from pDG-A-X series were tested by colony PCR, plasmid digestion and DNA sequencing. The results showed that two main types of deletion appeared, leading to excision of the first or the second gRNA expression cassette along with its promoter. It indicated that the recombination solely occurred between two identical promoters or scaffolds. Therefore, the replication slippage model for RecA-independent recombination was adapted to explain the phenomenon. During replication, pDG-A-X series generated the Types I or Type II slipped misalignment of the Okazaki fragment, which formed a loop within the lagging strand template to facilitate deletion (Fig. 3). However, the ratio between the two types of deletion couldn’t be measured, because MUT-1 and MUT-2 didn’t appear simultaneously in each recombination test of pDG-A-X series. Specific DNA sequences between DRs may form secondary structures and promote misalignment of one DNA strand on its complement [31]. It is likely that the 20-bp spacer sequences and the intervening sequences of paired gRNA cassettes have an influence on the type of deletion. In addition, the point mutations in the promoter and the insertion mutations in the scaffold may be attributed to the interruptions and errors of DNA replication induced by DRs.

According to the slipped misalignment model, expansion should be produced as efficiently as deletion [16]. However, the expansion of gRNAs could hardly be observed in this study. This agreed with the single strand annealing model which predicted that only deletion, instead of expansion, was efficiently produced in the presence of DRs separated by palindromes [16]. Although single strand annealing was inefficient in *E. coli* due to the rampant DNA degradation by the RecBCD, it suggested that the secondary structures of the intervening sequence could increase the frequency of deletion. Thus, the hairpin structure within the gRNA scaffold [1] may contribute to the domination of the deletion event in plasmid rearrangement.

We further investigated the effects of the transformation methods and culture media on plasmid recombination. Compared with chemical transformation, introducing the gRNA plasmid via electroporation significantly reduced its deletion frequency. The specific reasons on why transformation methods affected plasmid recombination events are not clear. However, based on the replication slippage mechanism, we inferred that the extent of cellular damage induced by different transformation approaches and their impacts on cellular processes could explain the difference in plasmid stability. During the chemical transformation, the growth of cells may be hindered, due to the loss of lipids, proteins and cell contents [32–34]. In contrast, a brief electrical pulse at appropriate voltage would cause only limited cell damage, and transformants are recovered at a frequency often much higher than that derived from other methods [35]. Therefore, it is likely that an aberrant metabolic state of the transformed cells, mutagenic replication or damaged DNA is prone to appear after chemical transformation [36]. In this case, the cellular replication processes could be greatly hindered, causing the pause of DNA polymerase and thus inducing the DRs-related recombination. Compared with LB medium, the increased amounts of yeast extract in TB medium provides abundant nutrient and growth factors, which promotes rapid plasmid propagation and prevents depletion of resources in cells [37]. As the pause of DNA polymerase is thought to be the first step in RecA-independent rearrangements [22], rapid DNA replication could prevent the nascent strand from translocating and pairing to other repeated regions. Therefore, the nutrient abundance of the medium may be the key factor on the stability of the plasmid containing DRs, and other rich media that promote rapid plasmid propagation could have the same effect as TB medium on the plasmid stability. As a result, the deletion frequency of pDG-A-100K decreased significantly when LB medium was replaced by TB medium.

Based on the replication model of RecA-independent recombination, the promoter of plasmid was further changed to avoid DRs-induced rearrangements. Although the disruption of promoter repeats could increase plasmid stability theoretically, but it was still difficult to obtain the correct plasmid by using Gibson Assembly method. According to the assembly mechanism of the method, the T5 exonuclease might overly process the DNA fragment, generating a prolonged single stranded overhang at the 3′ end. Therefore, it is likely that only one end of the backbone can be assembled correctly to the insert, whereas the other end is mismatched to the first repeated gRNA scaffold sequence in the insert, and thus leading to rearrangement of the gRNA plasmids. In this case, other assembly methods in vitro or in vivo that involved with exonuclease for terminal micro-homologous junction may face the same problem when used for the construction of paired gRNA plasmids. Furthermore, even if the correct plasmid could be obtained by certain assembly method, the plasmid stability would be compromised by the two identical gRNA scaffolds during DNA replication.

Changing ORI of a plasmid provides a convenient means of controlling the gene expression and can dramatically change the performance of the engineered system within a host [38–40]. However, changing the ORI had no effect on eliminating plasmid rearrangements in this study. The ORI only mediates the initiation of DNA replication but does not regulate the elongation rate of DNA polymerase [26, 41, 42]. Therefore, once replication initiates, DNA polymerase would move along the melted strand in a settled rate. No interaction between the ORI and the polymerase would occur to cause the pause or disassociation of the polymerase that is the prerequisites for the RecA-independent recombination.

If the presence of DRs could be eliminated, the paired-gRNA plasmid would be more stable. We further developed a RPGPs cloning strategy by changing the promoter of the second gRNA, the ORI and the direction of gRNA cassettes. The usage of two different promoters not only eliminated a pair of DRs, but also ensured the order of the two 20-bp spacers. Placing two gRNA cassettes in opposite directions then turned two 82-bp gRNA scaffolds into IRs. In general, IRs are often placed between DRs as intervening sequences to facilitate the misalignment between DRs [43–45]. It seems that IRs on pDG-R-X could hardly induce deletion without the participation of DRs. Thus, the pDG-R-X series showed 100% stability in this study. Moreover, even if IRs-mediated recombination could invert the intervening sequence under certain conditions [40], it would not jeopardize the expressions of gRNAs in pDG-R-X series. The RPGPs cloning strategy was also employed to construct paired-gRNA plasmids with ColE1 ORI. These plasmids showed the same 100% stability as that of the pDG-R-X plasmids in both plasmid construction and its propagation after re-transformation, but led to a high fatality rate in large genomic editing (data not shown). The high-copy-number ColE1 in this plasmid enhanced the expression of paired gRNAs and the ability of CRISPR/Cas9 system to generate DSBs, and therefore, cells were unable to repair the increased number of DSBs. Thus, a low-copy-number pSC101-based plasmid (pDG-R-X) would be more suitable to achieve large genomic editing in *E. coli*.

The genetic basis of the RecA-independent recombination was shown to be independent of RecBCD, RecET, RecF, RecG, RuvAB and other known recombination mechanisms in *E. coli*, when genome or plasmids were used as recombination substrates [16, 20]. Thus, other recombinases may play little role in pair-gRNA plasmids recombination. Moreover, RecA-independent recombination is enhanced by mutations that affect the DNA replication reliability, such as mutation of topisomerase III (*topB*), DNA polymerase I (*polA*), Exonuclease I (*sbcB*) and methyl-directed mismatch repair system (*mutS*, *mutL*, *mutH* and *Dam*) [16, 25]. Compared with other common strains used in this study, DB3.1 strain does not have the modification (methylation) system with the genotype of *m*_*B*_^−^ (Additional file 1: Table S1). The enhanced RecA-independent recombination in DB3.1 strain may be due to the absence of methyl-directed mismatch repair system. The increased plasmid deletion frequency in MG1655 could be induced by factors other than the activity of RecA. As a wild-type *E. coli* strain, MG1655 has the complex defense systems against exogenous DNA. For example, the nonsequence-specific DNA endonuclease I (*endA*) can cleave within double-stranded DNA (dsDNA) to produce short oligonucleotides [46]. MG1655 also possesses the functional restriction–modification systems to cleave incoming DNA if it has not been modified by a cognate or other appropriate methylase [47]. Therefore, the paired-gRNA plasmid introduced into MG1655 would be frequently cleaved by these endogenous defense systems and subsequently inducing slipped misalignment and deletion of the gRNA cassette. According to the above, the key factor in pair-gRNA plasmids recombination may be DNA replication errors or damages rather than other common recombinases. Although several specific commercial strains (e.g. Stbl3 or NEB stable) with the *recA13* or *recA1* genotype may be suitable for cloning unstable DNA in some cases, they cannot reduce the occurrence of the RecA-independent recombination. Moreover, even if isolation of plasmid containing repeat elements could be facilitated by these commercial strains, the plasmids would again suffer from the high-frequent rearrangement once introduced into the targeted hosts for metabolic engineering. Therefore, our RPGPs strategy which is not limited by host strains has a great potential to be applied for CRISPR/Cas9 systems in various *E. coli* strain with different genotypes.

In this study, we developed a simple but effective RPGPs cloning strategy to eliminate DRs-mediated plasmid recombination. Although the reversed-assembly cloning strategy may be limited to multiple genomic modification simultaneously, the stability of paired-gRNA plasmids is greatly enhanced, which can facilitate fast and high-efficiency multiple rounds of gene editing in *E. coli*.

## Conclusions

In summary, the deletion mechanism of DRs-involved paired-gRNA plasmids was investigated and a simple RPGPs cloning strategy for coexpressing paired gRNAs was developed by converting DRs to the more stable IRs. Using RPGPs can completely eliminate DRs-mediated recombination events, improve the performances of CRISPR systems that rely on paired gRNAs, and also facilitate other applications involving repeated genetic parts. This is a well-designed and illustrated technique with no special requirement, and therefore, it can be used by any biological lab easily.

## Methods

### Strains and cultivation conditions

All strains mentioned in this study were listed in Additional file 1: Table S1. *E. coli* DH10B and XL10-Gold are *recA1* strains expressing mutated RecA protein which has a G to A point mutation at position of the 482th base for reducing occurrence of nonspecific recombination in cloned DNA. Mach1-T1 Δ*recA1398* strain has truncated non-functional RecA protein, and DB3.1 Δ(*sr1*-*recA*) strain has no RecA protein. As a wild-type laboratory strain that has few genetic manipulations, MG1655 *recA*^+^ strain has the intact RecA protein. DH10B was used as a main cloning strain, and MG1655 was used in the genome editing experiments. Luria-Bertani (LB) broth (10 g/L tryptone, 5 g/L yeast extract, 10 g/L NaCl) was used for cell growth in all cases unless otherwise noted. Terrific Broth (TB) medium (12 g/L tryptone, 24 g/L yeast extract, 4 mL/L glycerol, 17 mM KH_2_PO_4_:72 mM K_2_HPO_4_ buffer solution) was also used for cell growth under some conditions. SOC medium (20 g/L tryptone, 5 g/L yeast extract, 0.5 g/L NaCl, 2.5 mM KCl, 10 mM MgCl_2_, 10 mM MgSO_4_, and 20 mM glucose) was used for cell recovery. Twenty g/L agar was supplemented if needed. Antibiotics were added at the following final concentrations: ampicillin, 100 μg/mL; kanamycin, 50 μg/mL. When appropriate, 20 mM l-arabinose were supplemented into media or cultures.

### Plasmids constructions

The plasmids used in this study were described in Additional file 1: Table S1. Target sequences of 100-kb fragment and all primers were given in Additional file 1: Table S2. pDG-A-X series, a derivative of pKB, contained ColE1 ORI and two identical J23119 promoters (http://parts.igem.org/Part:BBa_J23119) in the same direction to drive paired gRNAs. pDG-P-X series contained a J23119 promoter and a P_R_ promoter (http://parts.igem.org/Part:BBa_R0051) to drive paired gRNAs. pDG-S-X series, a derivative of pKS, contained pSC101 ORI and two identical J23119 promoters in the same direction for gRNAs expression. pDG-R-X series, a derivative of pKT, contained pSC101 ORI and two different promoters in the opposite direction for co-expression of paired gRNAs. The specific modular construction strategies of pDG-A-X and pDG-R-X series are shown in Figs. 1a and 5a, respectively. All of the paired-gRNA plasmids were constructed by using the Gibson Assembly method which can facilitate two overlapping DNA fragments to be assembled into a circular molecular via the concerted action of a 5′ exonuclease, a DNA polymerase and a DNA ligase [48]. For the PCR reaction, the 20-bp spacer sequences specific for two targeted loci were embedded in primers as a part of insert.

All the DNA fragments were PCR-amplified with Phusion polymerase (New England BioLabs). PCR products were gel purified, digested with *Dpn*I enzyme (Thermo Fisher Scientific) before assembly, which could eliminate the template plasmids. Gibson Assembly^®^ Master Mix were ordered from New England BioLabs. *Nde*I and *Cai*I enzymes (Thermo Fisher Scientific) were used to digest pDG-A-100K, while *Eco*32I and *Pst*I enzymes (Thermo Fisher Scientific) were used to digest pDG-R1-100K.

### Plasmid deletion assays

For each round of transformation, approximately one hundred colonies were formed on the LB plate (Amp) under the control. Twenty colonies on the LB plate (Amp) were selected randomly and verified by colony PCR in each experiment. The plasmid deletion frequency was defined as the proportion of colonies containing mutations among the 20 randomly picked colonies. Three biological replicates were tested for each group of experiments unless otherwise noted.

### Genome editing procedure

First, MG1655 containing p-P_BAD_-cas9 [28] electrocompetent cells were prepared. Then, specific plasmid pDG-R1-100K (100 ng) were added in each electroporation reaction. Bio-Rad MicroPulser was used for electroporation (0.1 cm cuvette, 1.80 kV). Cells after electroporation were immediately added into 1 mL SOC medium and recovered for 45 min prior to plating on LB (Amp + Kan) plate at 30 °C. A single colony was picked and inoculated into 0.5 mL SOC medium and cultured at 30 °C for 2 h. Then, 4.5 mL LB (Amp + Kan) medium were added to the cultures. After 1 h, l-arabinose (20 mM) was added, and the cultures were cultured for another 3 h before plating. A 10-μL aliquot of the cultures was plated onto a LB (Amp + Kan) plate containing l-arabinose, overnight at 30 °C. To analyze the mutation types of colonies, PCR products of the target locus were cloned and subjected to DNA sequencing.

## Supplementary information

**Additional file 1.** Additional Tables S1 and S2.

## Data Availability

All data generated or analyzed during this study are included in this published article and its additional file.

## Abbreviations

Amp: Ampicillin
CRISPR: Clustered regularly interspaced short palindromic repeats
DRs: Direct repeats
DSBs: The double-strand breaks
gRNAs: Guide RNAs
IRs: Invert repeats
Kan: Kanamycin
LB medium: Luria–Bertani medium
ORI: Origin of replication
PCR: Polymerase chain reaction
RPGPs: Reversed paired-gRNA plasmids
TB medium: Terrific Broth medium
