# Supplementary material for: Reversed paired-gRNA plasmid cloning strategy for efficient genome editing in Escherichia coli
Source: Microb Cell Fact. 2020 Mar 10;19:63. doi: 10.1186/s12934-020-01321-4 (PMC7063769; doi:10.1186/s12934-020-01321-4)
Supplement: Supplementary file 1 — Additional file 1. Additional Tables S1 and S2. [file 12934_2020_1321_MOESM1_ESM.docx]

**Additional Data**

**Tables**

**Table** **S1.** Strains and plasmids used in this study.

| **Strains or plasmids** | | **Descriptions** | **Source** |
| --- | --- | --- | --- |
| **Strains** | |  |  |
| *E. coli* DH10B | F^-^ λ^-^ *endA1 recA1 mcrA galU galK nupG rpsL* Δ*lacX74* Δ(*mrr-hsdRMS-mcrBC*) *φ80lacZ*Δ*M15 araD*Δ*139* Δ(*ara-leu*)*7697* (Sm^R^) | | Invitrogen |
| *E. coli* XL10-Gold | *endA1 recA1 thi-1 gyrA96 relA1 lac* Hte Δ(*mcrA*)*183* Δ(*mcrCB-hsdSMR-mrr*)*173* Tet^R^ F′[*proAB lacI^q^Z*Δ*M15 Tn10* (Tet^R^) Amy Cm^R^ ] | | Invitrogen |
| *E. coli* Mach1-T1 | F^-^ *endA1* Δ*recA1398 tonA φ80*(*lacZ*) Δ*M15* Δ*lacX74 hsdR*(*r_k_ ^-^*, *m_k_^+^*) | | Invitrogen |
| *E. coli* DB3.1 | F^-^ *endA1* Δ(*sr1-recA*) *gyrA462 mcrB mrr hsdS20*(*r_B_^-^*, *m_B_^-^*) *supE44 ara-14 galK2 lacY1 proA2 rpsL20* (Sm^R^) *xyl-5λ-leumtl1* | | Biomed |
| *E. coli* MG1655 | F^-^ λ^-^ *ilvG- rfb-50 rph-1* | | Our lab |
| **Plasmids** |  | |  |
| pKB | ColE1; J23119 promoter, a gRNA scaffold; Amp^R^ | | This study |
| pKI | ColE1; J23119 promoter, a gRNA scaffold; Amp^R^ | | This study |
| pPR | ColE1; P_R_ promoter, a gRNA scaffold; Amp^R^ | | This study |
| pKS | pSC101; J23119 promoter, a gRNA scaffold; Amp^R^ | | This study |
| pKT | pSC101; J23119 promoter, P_R_ promoter, two gRNA scaffolds; Amp^R^ | | This study |
| pDG-A-100K | ColE1; two J23119 promoters, two gRNAs; Amp^R^ | | This study |
| pDG-P-100K | ColE1; J23119 promoter, P_R_ promoter, two gRNAs; Amp^R^ | | This study |
| pDG-S-100K | pSC101; two J23119 promoters, two gRNAs; Amp^R^ | | This study |
| pDG-R-100K | pSC101; J23119 promoter, P_R_ promoter, two gRNAs; Amp^R^ | | This study |
| p-P_BAD_-cas9 | p15A; Cas9; Kan^R^ | | ^[1]^ |

**Table** **S2.** Target sequences of 100-kb fragment and all primers used in this study.

**Target sequences of 100-kb fragment.**

| **Spacer** | **Nucleotide sequence (5’-3’)** | **PAM** |
| --- | --- | --- |
| 100K-1 | gtaaaaccgaatactgccgg | tgg |
| 100K-2 | gaagtggctaaagagaacaa | cgg |

**Primers for the construction of paired-gRNA plasmids.**

| **Primer Name** | **Primer sequence (5’-3’)** |
| --- | --- |
| pKB backbone _F | gttttagagctagaaatagc |
| pKB backbone_R | actagtattatacctaggac |
| pDG-A-100K insert_F | gtcctaggtataatactagtgtaaaaccgaatactgccggttttagagctagaaatagc |
| pDG-A-100K insert_R | gctatttctagctctaaaacttgttctctttagccacttcactagtattatacctaggac |
| pDG-P-100K insert_F | gtcctaggtataatactagtgtaaaaccgaatactgccggttttagagctagaaatagc |
| pDG-P-100K insert_R | ctatttctagctctaaaacttgttctctttagccacttcgcaaccattatcaccgcca |
| pKS backbone _F | gttttagagctagaaatagc |
| pKS backbone_R | actagtattatacctaggac |
| pDG-S-100K insert_F | gtcctaggtataatactagtgtaaaaccgaatactgccggttttagagctagaaatagc |
| pDG-S-100K insert_R | gctatttctagctctaaaacttgttctctttagccacttcactagtattatacctaggac |
| pKT backbone_F/R  pDG-R-100K insert_F  pDG-R-100K insert_R | gttttagagctagaaatagc  tatttctagctctaaaacccggcagtattcggttttacactagtattatacctagg  tatttctagctctaaaacttgttctctttagccacttcgcaaccattatcaccgcc |

To clone pDG-A-100K, primers pKB backbone _F/pKB backbone _ R were used to amplify pKB, and primers pDG-A-100K insert_F/pDG-A-100K insert_R were used to amplify pKI. The PCR products were assembled through Gibson Assembly method ^[2]^.

To clone pDG-P-100K, primers pKB backbone _F/pKB backbone _ R were used to amplify pKB, and primers pDG-P-100K insert_F/pDG-P-100K insert_R were used to amplify pPR. The PCR products were assembled through Gibson Assembly method ^[2]^.

To clone pDG-S-100K, primers pKS backbone _F/pKS backbone _ R were used to amplify pKS, and primers pDG-S-100K insert_F/pDG-S-100K insert_R were used to amplify pKI. The PCR products were assembled through Gibson Assembly method ^[2]^.

To clone pDG-R-100K, primers pKT backbone _F/pKT backbone _ R were used to amplify pKT, and primers pDG-R-100K insert_F/pDG-R-100K insert_R were used to amplify pKT. The PCR products were assembled through Gibson Assembly method ^[2]^.

**Screening and sequencing primers of paired-gRNA plasmids.**

| **Primer Name** | **Primer sequence (5’-3’)** |
| --- | --- |
| F1 | ttgacagctagctcagtcct |
| R1 | ctctgctaatcctgttaccag |
| F2 | taacaccgtgcgtgttgact |
| R2 | ttggtggttgataagcgagg |
| F3 | gatcactacttcgcactagt |
| R3 | taatactagtgtaaaaccga |
| F4 | taatggttgcgaagtggcta |
| gRNA-Sequencing_1 | aacgcggcctttttacggttc |
| gRNA-Sequencing_2  gRNA-Sequencing_3 | tccccgaaaagtgccacctg  actacacgatgctttaactg |

For screening of pDG-A-100K, primers F1/R1 were used. Primer gRNA-Sequencing_1 was used for DNA sequencing.

For screening of pDG-P-100K, primers F1/R1 or F2/R1 were used. Primer gRNA-Sequencing_1 was used for DNA sequencing.

For screening of pDG-S-100K, primers F1/R2 were used. Primer gRNA-Sequencing_2 was used for DNA sequencing.

For screening of pDG-R1-100K, primers F3/R3 and F4/R2 were used respectively. Primers gRNA-Sequencing_2 and gRNA-Sequencing_3 were used for DNA sequencing.

**Screening and sequencing primers for 100-kb genome editing.**

| **Primer Name** | **Primer sequence (5’-3’)** |
| --- | --- |
| F5 | gcaaatgttgcccacgaatg |
| R5 | ggacgaacgtaccgctatg |
| F6 | cggtcgatatgcggatgtat |
| R6 | tctcctgctgcaggattttg |
| F7 | gacaacaagcccctgattac |
| R7 | gaactggggaggcgactatt |

**Reference**

[1] Chaoyong Huang, Tingting Ding, Jingge Wang, Xueqin Wang, Liwei Guo, Jialei Wang, Lin Zhu, Changhao Bi, Xueli Zhang, Xiaoyan Ma, Y.-X. Huo, *Applied Microbiology and Biotechnology* **2019**, *103*, 8497–8509.

[2] D. G. Gibson, E. Al, *Nature Methods* **2009**, *6*, 343.
